# Characteristics and Outcome Analysis for Intensive Care Patients Undergoing Decompressive Laparotomy for Abdominal Compartment Syndrome: Impact of Extracorporeal Membrane Oxygenation Support

**DOI:** 10.3390/jcm12237403

**Published:** 2023-11-29

**Authors:** Christine Nitschke, Marco Schulte, Jakob R. Izbicki, Thilo Hackert, Stefan Kluge, Christoph Burdelski, Kai Bachmann

**Affiliations:** 1 Department of General, Visceral and Thoracic Surgery, University Hospital Hamburg-Eppendorf, 20246 Hamburg, Germany; izbicki@uke.de (J.R.I.); t.hackert@uke.de (T.H.);; 2 Department of Intensive Care, University Hospital Hamburg-Eppendorf, 20246 Hamburg, Germanys.kluge@uke.de (S.K.); cburdelski@uke.de (C.B.)

**Keywords:** abdominal compartment, ECMO, laparotomy, ICU

## Abstract

(1) Background: Abdominal compartment syndrome (ACS) is a life-threatening situation and is associated with high mortality in the intensive care unit (ICU). Decompressive laparotomy represents the last therapeutic option. This cohort study aims to optimize the selection of ICU patients suffering from ACS who benefit from decompressive laparotomy. (2) Methods: All available data from adult patients treated at the 12 ICUs of a university hospital between 2011 and 2019 were included. Outcome parameters for patients with and without extracorporeal membrane oxygenation (ECMO) were compared. (3) Results: 207 ICU patients with ACS undergoing surgery were identified. Laparotomy resulted in immediate improvement of organ functions in 15% of patients, who then survived more frequently. The overall mortality rate in our cohort was 69%. The group of ECMO patients—including va- and vv-ECMO—showed significantly less organ function improvement and a higher mortality rate of 79% compared to a better postoperative improvement and a lower mortality rate of 62% in non-ECMO patients. (4) Conclusions: There are ICU patients who benefit from decompressive laparotomy—nevertheless, mortality is high. Non-ECMO patients have a better prognosis than ECMO patients. Our findings can support clinical decision-making on emergency surgery and the development of future guidelines.

## 1. Introduction

Intra-abdominal hypertension (IAH) is common in critically ill patients with cardiac, pulmonary, or septic underlying diseases, acute respiratory distress syndrome (ARDS), trauma, or after surgery. It often leads to severe organ dysfunctions [1,2,3,4,5] and is an independent predictor for mortality [1,2,3,4,5]. When IAH is developing into an ACS, organ failure occurs by definition.

The term abdominal compartment syndrome (ACS) is characterized by a tensely distended abdomen, elevated intra-abdominal pressure (>20 mmHg) and high peak airway pressures, inadequate ventilation with hypoxia and hypercapnia, and a disturbed renal function [1,5]. If medical therapy fails to reduce intra-abdominal pressure via fluid management or minor invasive methods (such as percutaneous drainage of fluid collections or nasogastric decompression, etc.), decompressive laparotomy is the only treatment option [6,7,8,9,10]—if not performed, death has to be hypothesized. A surgical bedside evaluation of the patient is necessary for decision-making on performing surgery. In most cases, surgery has to be performed as a rescue bedside surgery procedure in the ICU since most patients are too unstable for transportation to the operating room (OR) [8,11]. Organ functions often show improvement after performing decompressive laparotomy, although mortality among patients with ACS remains high [4,7,12,13,14]. 

In particular, severely ill patients on extracorporeal life support (ECLS), like extracorporeal membrane oxygenation (ECMO) support, frequently develop ACS, which further complicates their already critical clinical course [6,15]. Prevalent underlying indications for veno-arterial (va)-ECMO are myocardial infarction, extracorporeal cardiopulmonary resuscitation (eCPR), and postcardiotomy syndrome. The indication for veno-venous (vv)-ECMO support is mainly an ARDS [6,12,16,17,18]. The mortality rate for ECLS patients is generally high even without ACS (36–60%) [6,12,16,17,18]. In ACS patients, mortality rates were observed to be higher for ECLS patients than for non-ECLS patients and were associated with higher age, comorbidities, and a high Simplified Acute Physiology Score (SAPS) II [6,15].

Since knowledge about impact factors on the outcome of patients with an ACS undergoing decompressive surgery is still poor, further investigation is required to identify those patients who would mostly benefit from surgery [6,12,18,19]. 

Therefore, this cohort study aims to evaluate the benefit of an emergency decompressive laparotomy in relation to underlying medical conditions (such as ECLS), looking at postoperative organ function improvements and survival in ICU patients suffering from ACS.

## 2. Materials and Methods

### 2.1. Ethical Approval

The local ethical committee approved the study (2022-100971-WF).

### 2.2. Setting

The Department of Intensive Care Medicine at the University Hospital Hamburg-Eppendorf includes 12 ICUs with 140 beds. Besides units being specialized for surgical, medical, and neurological critical ill patients, some units are specialized for ECLS in case of ARDS or cardiac failure.

### 2.3. Study Design

In this cohort study, we analyzed available data from anonymized adult patients being treated at the Department of Intensive Care Medicine of the University Hospital Hamburg-Eppendorf for ACS between 2011 and 2019. For data collection, the following items were included: ICU parameters and administrative data from patients diagnosed with ACS who received a decompressive laparotomy. Patients who were under the age of 18 years were excluded, and data from patients with repetitive decompressive laparotomies during one in-hospital stay were analyzed only for their first occurrence of ACS with consecutive decompressive laparotomy.

### 2.4. Abdominal Compartment Syndrome Diagnosis and Treatment

The requirement for the diagnosis of ACS was defined as abdominal distension with elevated intra-abdominal pressure (>20mmHg = IAH) and disturbed organ functions. The measurement of intra-abdominal pressure was conducted in accordance with the existing literature [8]. The decompressive laparotomy was performed via a vertical, midline, full-thickness abdominal incision from the xyphoid process to the pubis and with the use of electrocautery for minimized bleeding, according to the literature [20]. Other decompression techniques were not included. As abdominal closure techniques, Bogota bags or Vicryl nets were used for temporary abdominal closure.

### 2.5. Underlying Causes of Abdominal Compartment Syndrome and Postoperative Outcomes

For each patient, we reported age, gender, the underlying cause of disease, as well as whether the patient was on ECLS and had an additional ARDS. We differentiated between primary ACS causes, such as bleeding, and secondary ACS, such as cardiac, pulmonary, or septic causes. Furthermore, we analyzed whether resuscitation took place prior to surgery, whether the surgery was performed at the ICU or in the OR, and whether ascites or intra-abdominal comorbidities were present at surgery. As a primary outcome, immediate postoperative improvement of organ functions was analyzed—characterized by postoperative changes in respiratory parameters: oxygenation index (paO2/FiO2), SpO2, FiO2, respiratory pressure, and circulatory parameters: lactate, urine production, catecholamine dosage, and bladder pressure, whereas mortality was the secondary outcome.

The measured improvement of organ functions was based on postoperatively observed changes in the FiO2, SpO2, lactate levels, vasopressor support, parameters of mechanical ventilation, and urinary output. 

### 2.6. Statistical Analysis

Data were analyzed using SPSS (IBM Corporation, New York, NY, USA, 19th version). A multivariate analysis was used for preoperative parameters, applying a logistic regression analysis. The chi-squared test was used to express proportions and compare categorical data. The Student’s *t*-test was used for continuous and normal distributed variables. A 95% confidence interval and a significance level of 0.05 were chosen to be significant.

## 3. Results

### 3.1. Patient Characteristics

In total, 207 patients who underwent decompressive laparotomy for ACS at the University Hospital Hamburg-Eppendorf between 2011 and 2019 were identified. The median age of patients was 58 years, 51% were male and 49% were female. These patients showed a broad variety of underlying diseases (Table 1).

Out of all 207 ACS patients, 71 patients (34%) were on ECLS (23 on vv-ECMO-support and 48 patients on va-ECMO-support). Further baseline characteristics can be found in Table 2.

Organ functions improved postoperatively in 53% of these patients: 15% showed an immediate recovery within one hour, and 38% showed a slow recovery of organ functions within six hours after surgery. 

### 3.2. Mortality

The ICU mortality was 69.1% (*n* = 143) (median survival: 2 days; range 0–126 days). The 24 h mortality was 31% (*n* = 62), and the 28-day mortality was 61% (*n* = 121). Generally, those patients who did not show postoperative organ function improvement died (*p* < 0.001). 

### 3.3. Predictors for Mortality

Significant differences in mortality were observed for several factors (Table 3). 

One of the most prominent results concerned patients who did not recover after surgery. These patients showed an almost 99% mortality. On the other hand, patients with immediate or at least slow improvement after surgery showed significantly lower mortality (36.7/45.3%) (*p* < 0.001). 

A markedly higher mortality was also observed for patients with ascites present at surgery (*p* = 0.072). All patients (*n* = 22) with additional pathologic findings, such as mesenteric infarction or peritonitis, that underwent decompressive laparotomy in the ECLS group died (*p* < 0.001).

To identify independent preoperative—prognostic factors for survival that allow identifying patients that will benefit from the laparotomy—a multivariate regression analysis on the preoperative parameters was added. Neither age, sex, status post-resuscitation, nor the underlying disease was found to be an independent prognostic factor, but patients on ECLS (OR 2.564; *p* = 0.009) and too unstable patients being operated at the ICU bedside without transport possibilities to the OR (OR 2.749; *p* = 0.008) have been found to be independent prognostic factors for mortality. 

### 3.4. Abdominal Compartment Syndrome in Extracorporeal Life Support and Non Extracorporeal Life Support Patients

Laparotomy of ECLS patients resulted in an immediate improvement of organ functions in 11% of patients. Survival was significantly associated with those patients who showed an immediate recovery (14% (30/207) of all patients) after decompressive laparotomy (*p* = 0.001). In total, 18% (25/136) of non-ECLS patients showed an immediate recovery, compared to only 7% (5/71) of ECLS patients (4/48 va-ECMO patients and 1/23 vv-ECMO patients). There was no significant difference in recovery after decompressive laparotomy between va- and vv-ECMO patients. 

Supplementary Table S1 underlines the postoperative changes in organ function parameters comparing ECLS and non-ECLS patients who underwent surgery. For the ECLS and non-ECLS group, a significant decrease in lactate after 24 h, a decrease in the need for catecholamines, and a postoperative decline in bladder pressure were observed. Also, in non-ECLS patients, a significant decrease in the maximum inspiration pressure was reached postoperatively, whereas significant increases in compliance and minute respiratory volume were observed in all groups.

**Figure 1:**
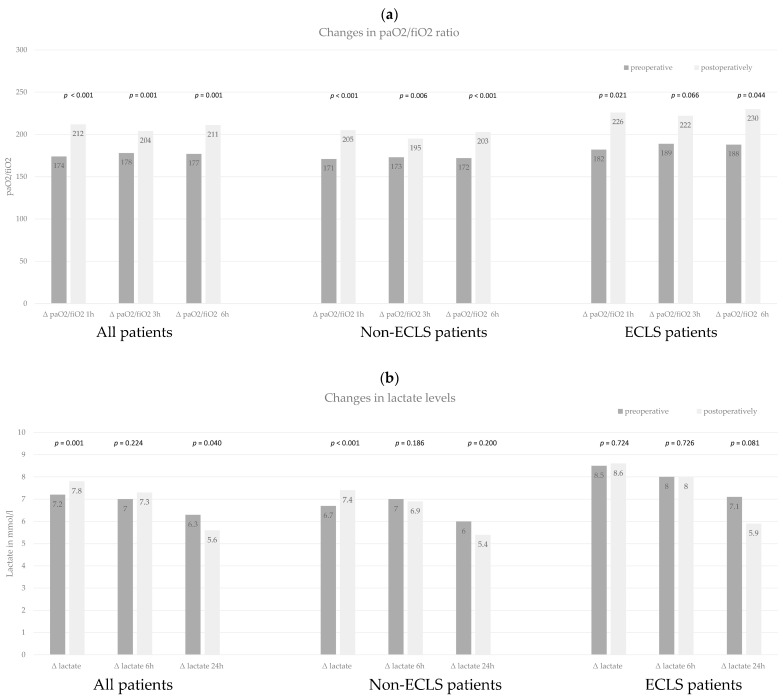
(**a**) Paired *t*-test for mean comparison of paO2/fiO2 ratio at different time points. We compared the preoperatively measured paO2/fiO2 ratio to postoperative values at 1 h, 3 h, and 6 h after surgery. This analysis was performed for all patients, non-ECLS patients, and ECLS patients. (**b**) Paired *t*-test for mean comparison of lactate levels at different time points. We compared the preoperative measured lactate levels to postoperative values directly after surgery and 6 h and 24 h after surgery. This analysis was performed for all patients, non-ECLS patients, and ECLS patients.

We also observed significant increases in the postoperative paO2/fiO2 ratio at all time time-points after surgery (at 1 h, 3 h, and 6 h, postoperatively) compared to preoperative values in the entire patient cohort and in non-ECLS patients (all *p* < 0.006), and for ECLS-patients at 1 h and 6 h after surgery (*p* = 0.021; *p* = 0.044) (Figure 1a and Supplementary Table S2). 

For the postoperative changes in the mean lactate levels, we observed a significant postoperative rise of lactate within the first hour after surgery in the entire patient cohort and non-ECLS patients (*p* = 0.001; *p* < 0.001) (Figure 1b and Supplementary Table S2). In the following hours postoperatively, a decrease in the mean lactate levels was observed for the entire cohort, non-ECLS patients, and ECLS patients—with a significant decrease compared to preoperative paired values in the entirety of patients at 24 h after surgery (*p* = 0.040).

### 3.5. Abdominal Compartment Syndrome in Acute Respiratory Distress Syndrome Patients

Of all 207 ACS patients, 21% of patients (*n* = 43) were additionally suffering from ARDS—including both vv-ECMO patients and non-ECMO patients. Most patients with ARDS showed improvement of organ functions directly after surgery (Table 4): the oxygenation index, pulmonary compliance, and respiratory volume per minute increased in the majority of patients (61.3% oxygenation index increase, 78.6% pulmonary compliance increase, 68.4% increase in respiratory volume), and decreased lactate and catecholamine levels were also observed in relevant numbers of patients (30.6% had decreased lactate and 23.7% a decrease in catecholamine levels). In Table 4, the increase in values was compared to equal values and to decreased values as categorical data via descriptive statistics.

In vv-ECMO patients with ARDS, improvement in SpO2 oxygenation after decompressive laparotomy was more prominent (with an increase of 3.6% of the metric SpO2 value on average within one hour after surgery) compared to va-ECMO patients without ARDS, for whom the SpO2 value increased only by 0.8%, which might be caused by an increased oxygen consumption due to the postoperatively improved abdominal perfusion. Although not significantly, average postoperative lactate values increased only in the group of va-ECMO patients, whereas they showed a decrease in vv-ECMO patients with ARDS. 

Beneath the slightly better recovery of organ functions, there was no difference in survival observed for vv- vs. va-ECMO, but vv-ECMO patients survived significantly longer after surgery (17 vs. 5 days on average before death; *p* = 0.041).

The subgroup of ECMO patients within our cohort of ACS patients showed a significantly higher ICU mortality in comparison to non-ECMO patients (83.1% (*n* = 59/71) vs. 61.8% (*n* = 84/136) (*p* = 0.002)). Nevertheless, even on ECMO support, 17% of patients were rescued by performing a decompressive laparotomy. There was no significant difference in survival between va- and vv-ECMO patients. Those who survived were discharged after 64 days on average (range: 17–178 days).

### 3.6. The Prediction of Organ Function Improvement and Mortality

Changes in organ function were observed immediately, after 6 h, and after 24 h following surgery, but the most prominent results were observed immediately.

For survivors, improvements were noted for postoperative changes in bladder pressure, spO2, fiO2 oxygenation, respiratory pressure, and catecholamine support—but there was no significant difference observed between those who died and those who survived.

**Figure 2:**
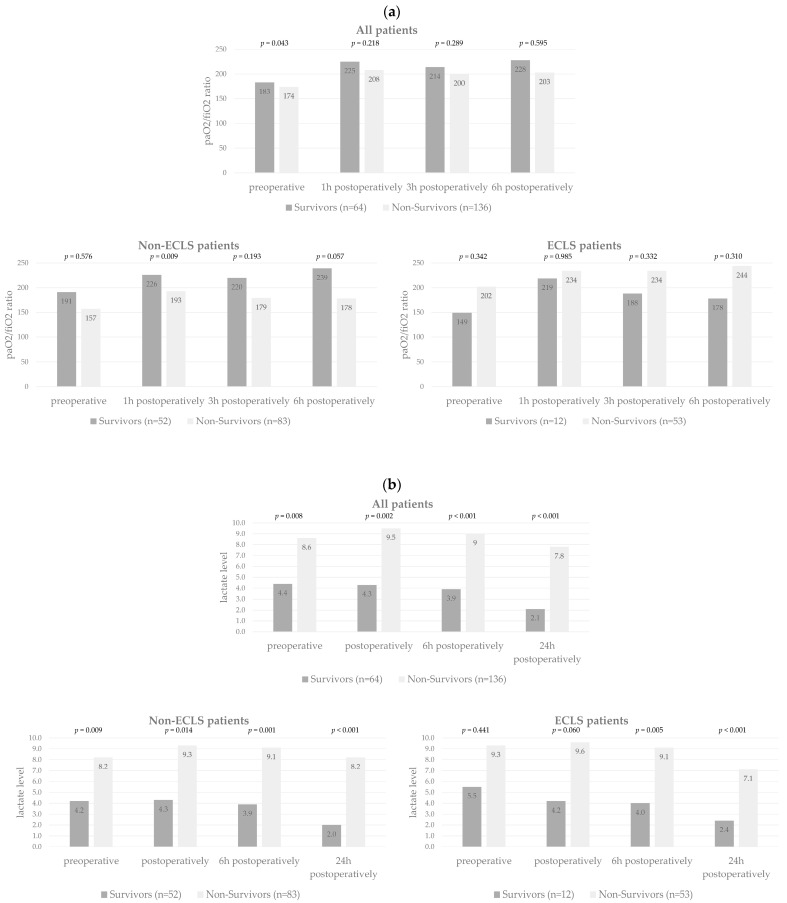
(**a**) *T*-test to compare mean paO2/fiO2 ratio for survivors and non-survivors at different time points for all patients, non-ECLS patients, and ECLS patients. (**b**) *T*-test to compare mean lactate levels for survivors and non-survivors at different time-points for all patients, non-ECLS patients, and ECLS patients.

We also observed a significant difference in the preoperative paO2/fiO2 ratio between all survivors and non-survivors (*p* = 0.043) and also for non-ECLS patients at the one-hour time-point after surgery (*p* = 0.009) (Figure 2a and Supplementary Table S3). 

We also observed significantly lower mean lactate levels for survivors compared to non-survivors of the entire study cohort as well as of non-ECLS patients at all time points (preoperatively, postoperatively, at 6 h, and at 24 h postoperatively) (all *p* < 0.014), and for ECLS-patients at 6 and 24 h postoperatively (*p* = 0.005 and *p* < 0.001) (Figure 2b and Supplementary Table S3).

In non-ECLS patients, a significantly higher mortality rate was observed for patients with intra-abdominal comorbidities or ascites present at surgery (*p* = 0.016) and for those not recovering after decompressive laparotomy (*p* < 0.001). Non-ECLS patients who were older than the median age of 58 showed significantly more often no recovery after decompressive laparotomy (*p* = 0.006).

Although the age of ECLS patients had no significant impact on any outcome parameter, survivors were younger on average than those who died (mean: 48 vs. 53 years), and those younger ECLS patients (less than the median age of 58 years) who died were alive for a longer period after surgery than older ECLS patients (12 vs. 5 days), showing at least a temporary improvement of oxygenation parameters.

## 4. Discussion

According to our results, predictors for mortality in ACS are the absence of immediate organ function recovery after surgery, preoperative ECLS, ascites or intra-abdominal comorbidities, and a postoperative increase in lactate. Particularly striking is the significant difference between the survival rates of 17% in ECLS patients and 38% in non-ECLS patients (*p* = 0.002).

Previous studies showed that laparotomy on patients with ACS led to a significantly reduced intra-abdominal pressure and improved oxygenation and urinary output and should, therefore, be recommended for patients with ACS [12,21]. We also observed improved organ functions (including SpO2, FiO2, and respiratory pressure) and reductions in intra-abdominal pressure (from 25 to 16 mmHg) following surgery, which is within the described range of bladder pressure reduction from previous studies [7,22,23]. Non-significant observations can be explained by incomplete data because of missing or undocumented values in the analyzed data, leading to smaller sample sizes for some of the analyzed parameters [24]. All in all, we noted significantly more often an immediate or slow improvement of organ function scores for survivors, whereas patients who died did not improve—which is in accordance with existing literature [12]. 

We observed that patients with ARDS and ACS especially benefitted from decompressive laparotomy, with improved respiratory functions in most patients and decreased lactate and catecholamines in many patients. 

Also, vv-ECMO patients suffering from ACS with an underlying ARDS showed better postoperative organ recovery, SpO2 improvement, and significantly longer survival compared to va-ECMO patients. In particular, these patients seem to benefit from emergency decompressive surgery. There is still a need for further investigation and knowledge in the field of ARDS and ACS [18]. 

Despite the high mortality rates among patients suffering from ACS who received a decompressive laparotomy, our results underline that there are predicting factors for survival [7,13,22,23,24,25]. 

We found significant differences in the survival rates of patients with ACS on ECLS and non-ECLS ACS patients. When analyzing our results for evaluating the benefit of decompressive laparotomy in ACS patients, we should, therefore, draw conclusions for patient groups separately. The literature describes an average mortality rate of 50% for adult ACS patients undergoing decompressive laparotomy [7,13,22,23,24]. This is less than the observed average mortality of 69% in our study, although our observed mortality rate is still in the range of mortality rates from previous studies [7,25]. This bias can be explained by differences in decision-making on surgery and the timing of surgery at different centers [7]. In the existing literature, survivors were younger, while in our study, age was not found to be a significant predictor for mortality—although we observed that younger patients had an immediate recovery of organ functions significantly more often. 

The observed initial lactate increase after decompressive surgery goes timely along with the reperfusion of intra-abdominal organs—and in this context, initial lactate release is an expected phenomenon, while the following decrease in lactate level shows the positive effect of performed surgery [26]. Furthermore, the observed significant increase in postoperative lactate in those patients who died compared to the survivors is also in accordance with previous studies of hyperlactatemia and low lactate clearance predicting poor clinical outcomes in critically ill ICU patients [27]. In these patients with extremely high ICU mortality, the benefit of continuing the ICU therapy should, therefore, be evaluated closely [27].

For vv- and va-ECMO patients suffering from ACS, our observation of a higher mortality rate compared to non-ECMO ACS patients (83% vs. 62%) is in accordance with the existing literature—as other studies describe mortality rates of 69% and 73% for ECMO patients with ACS and the need for an emergency decompressive laparotomy [6,15]. Furthermore, previous studies described that mortality among ECLS patients did not significantly differ for those with and without ACS (73% vs. 65%)—as mortality was associated with the pre-existing severity of the underlying diseases and older age [6]. They also classify ACS as an occasionally occurring, possible complication during ECLS, mostly being a secondary ACS [6,9]. This is in accordance with our observation that there is a significant association of ACS in ECLS patients. Another common observation in accordance with previous studies is that ECLS patients do not show large amounts of ascites, whereas present ascites in our non-ECLS patients were significantly associated with mortality [6]. Furthermore, older patients on vv- and va-ECMO support showed an extremely high risk of mortality and did not improve immediately after decompressive laparotomy. Thus, their indication for emergency surgery should be critically discussed, taking the underlying diseases into account for good ethical treatment. Vv- and va-ECMO patients with intra-abdominal pathology, besides the increased intra-abdominal pressure, will not benefit from decompressive laparotomy.

It has been previously discussed in the literature that surgical decompression—although being the definitive therapy for primary ACS (such as bleeding)—may not be the treatment of choice for every secondary ACS (especially for most vv- and va-ECMO patients) [7,28]. For the latter patients, the indication for an emergency surgery should, therefore, be an individual decision. To balance the benefits against possible adverse events of surgery (such as hemorrhagic complications on prolonged ECLS support), previous studies concluded that performing a decompressive laparotomy in ECLS patients did not increase the overall mortality—therefore, the benefit for those 17% of ECLS patients who survive after a decompressive laparotomy should always be taken into account and be balanced against the risk of complications—and a rescue surgery should be indicated, if appropriate [6,28,29]. 

All in all, an accountable percentage of non-ECMO (38%) and even severely ill vv- and va-ECMO patients (17%) can be rescued by undergoing an emergency laparotomy in ACS.

The wide variety of underlying diseases within our patient cohort needs to be addressed as a main limitation of this study since patients with cardiogenic shock, ARDS, intra-abdominal comorbidities, and septic shock were analyzed together. Regarding postoperative improvement of organ functions, as a primary outcome, ARDS patients were particularly identified to benefit from decompressive laparotomy. Regarding mortality, as a secondary outcome, intra-abdominal comorbidities were identified as predicting factors. Ascites, in particular, were predictors of mortality. There are multifactorial causes of ascites in our study population of ICU patients, such as a positive fluid balance, poor venous drainage, hepatic failure, and hypalbuminemia during the ICU course [30,31,32]. 

Nevertheless, the wide variety of underlying diseases was not identified as an independent prognostic factor for mortality in the performed multivariate analysis. This underlines that a joint analysis of the selected variety of underlying diseases in our cohort is adequate to address treatment decision-making in ACS patients in relation to their risk of mortality.

According to our knowledge, this is the biggest cohort study on emergency decompressive laparotomy in ACS patients, with *n* = 207 patients. In total, 31% of all ACS patients who underwent an emergency decompressive laparotomy survived. In contrast, if the indication for decompressive surgery was right, a mortality rate of nearly 100% had to be assumed. Therefore, the found predictors for survival in ACS patients (non-ECLS patients, stability for transport into the OR—which means an indication for surgery in time, non-cardiac cause, or an underlying ARDS)—can serve for individual preoperative decision-making and maybe for establishing a point-based score for decision-making on selecting who will benefit from surgery [6,7,12,25,33]. Kidney failure was interpreted as the first clinical sign of abdominal compartment syndrome.

The identified predictors for mortality, like intra-abdominal comorbidities, no immediate recovery of organ functions after surgery, and a persistent, postoperative increase in lactate, could also be helpful for the postoperative decision-making of physicians on withdrawing or continuing life-sustaining therapy in critical care [27,34].

## 5. Conclusions

This study confirmed decompressive laparotomy to be helpful in ACS patients, improving several parameters, especially lactate levels and paO2/fiO2 ratio. As the understanding of which ACS patients mostly benefit from decompressive laparotomy—in the form of immediate improvement of organ functions and reduced mortality—is still incomplete, a better selection of patients could lead to better clinical outcomes after surgery. Because of the correlation of higher lactate levels to mortality, this parameter must be measured and may influence decision-making processes in this clinical emergency situation of ACS. 

Therefore, our predicting factors for survival and mortality can serve for individual perioperative decision-making. They could also serve as a base for further clinical studies leading to the development of guidelines for the treatment of ACS patients.

## Figures and Tables

**Table 1:** Detailed description of underlying diseases.

Underlying Diseases	Total Number of Patients *n* = 207 (%)
1. Cardiogenic shock	48 (23%)
2. ARDS	40 (19%)
3. Postoperative abdominal hypertension	37 (18%)
4. Peritonitis	24 (12%)
5. Abdominal bleeding	17 (8%)
6. Pancreatitis	15 (7%)
7. Septic shock	14 (7%)
8. Others	12 (6%)

**Table 2:** Baseline characteristics of the included *n* = 207 patients (#: incomplete data).

Baseline Characteristics		Number of Individuals (*n*)	Percentage (%)
Age	<58 years	103	49.8
	≥58 years	104	50.2
Gender	Female	101	48.8
	Male	106	51.2
Prior mechanical ventilation #	No	18	9.2
	Yes	178	90.8
Prior vasopressor #	No	11	6.3
	Yes	163	93.7
Acute kidney injury #	No	81	46.6
	Yes	93	53.4
ECMO support	No	136	65.7
	Vv-ECMO	23	11.1
	Va-ECMO	48	23.2

**Table 3:** Factors associated with a marked difference in mortality in the 207 analyzed ACS patients (x: number of patients who died out of the number of total patients (*n*) with associated factor; mortality rate: % = x/*n*; *p* value (*: incomplete data)).

Abdominal Compartment		Mortality		*p* Value
		x/*n*	%	
Previous resuscitation *	yes	59/71	83.1	0.001
	no	73/122	59.8	
ECLS	yes	59/71	83.1	0.002
	no	84/136	61.8	
Surgical setting *	ICU	91/113	80.5	<0.001
	OR	45/86	52.3	
Ascites present at surgery *	yes	89/120	74.2	0.072
	no	46/75	61.3	
Recovery after surgical intervention *	immediately	11/30	36.7	<0.001
	slowly	34/75	45.3	
	none	91/92	98.9	
Total *n* = 207	Total deaths	143/207	69.1	

**Table 4:** Postoperative improvement of organ functions in acute respiratory distress syndrome patients (*n* = 43).

Postoperative Improvement of Organ Functions	Percentage of ARDS Patients with Indicated Improvement of Organ Functions (Including Number of Patients with Available Data Immediately after Surgery)
Increased oxygenation index (paO2/FiO2)	61.3% (19/31)
Increased lung-compliance	78.6% (11/14)
Increased respiratory volume per minute	68.4% (26/38)
Decreased lactate level	30.6% (11/36)
Decreased doses of catecholamines (µg/kgKG/min)	23.7% (9/38)

## Data Availability

Data is available upon request.

## Supplementary Materials

The following supporting information can be downloaded: https://www.mdpi.com/article/10.3390/jcm12237403/s1. Table S1: Postoperative changes in organ function parameters for all. ECMO and non-ECMO patients. Table S2: Paired *t*-test for mean comparison of paO2/fiO2 ratio and lactate levels at different time points. We compared the preoperatively measured paO2/fiO2 ratio and lactate levels to postoperative values directly at different time points. This analysis was performed for all patients, non-ECLS patients, and ECLS patients. Table S3: *T*-test to compare mean paO2/fiO2 ratio and lactate levels for survivors and non-survivors at different time points for all patients, non-ECLS patients, and ECLS patients.
