# Supplementary material for: Characteristics and Outcome Analysis for Intensive Care Patients Undergoing Decompressive Laparotomy for Abdominal Compartment Syndrome: Impact of Extracorporeal Membrane Oxygenation Support
Source: J Clin Med. 2023 Nov 29;12(23):7403. doi: 10.3390/jcm12237403 (PMC10707647; doi:10.3390/jcm12237403)
Supplement: Supplementary file 1 [file jcm-12-07403-s001.zip › jcm-2670236-supplementary.pdf]

Supplementary Table S1: Postoperative changes of organ function parameters for all patients, ECMO patients, and non-ECMO patients.

(Mean. Standard error (SE). p value) *(NA in µg/kgKG/min; oxygenation index (Horovitz) in mmHg; lactate in mmol/l; pmax and PEEP in mbar)

	All patients			Non-ECMO			ECMO (all)		
	Mean	SE	p	Mean	SE	p	Mean	SE	p
**catecholamine** dose preoperatively*	1.1	0.2	+	0.9	0.1	+	1.4	0.6	+
" directly afte surgery	1.3	0.3	0.124	1	0.1	0.146	2.1	1	0.236
catecholamine dose preoperatively*	1.1	0.2	-	0.9	0.1	-	1.6	0.8	-
" 6 hours after surgery	0.8	0.1	0.181	0.77	0.1	0.219	1	0.2	0.315
**bladder pressure** preoperatively (mmHg)	25.6	1.5	-	24.2	1.8	-	30.1	1.6	-
" " directly after surgery	16.4	1.2	**<0,001**	15.7	1.4	**<0,001**	18.8	1.6	**0.001**
**Pmax** preoperatively	28.1	0.6	-	28	0.8	-	28.2	1.1	+
" directly after surgery	27.1	0.5	**0.03**	26.3	0.6	**0.005**	28.6	0.8	0.486
Pmax preoperatively	28.2	0.7	-	28	0.8	-	28.4	1.1	-
" 3 hours after surgery	25.5	0.7	**<0,001**	24.7	0.8	**<0,001**	27.2	1.2	0.294
Pmax preoperatively	27.9	0.6	-	27.7	0.8	-	28.4	1.1	-
" 6 hours after surgery	24.4	0.7	**<0,001**	22.9	0.9	**<0,001**	27.3	1.2	0.37
**PEEP** preoperatively	10.6	0.4	+	10.6	0.5	+	10.5	0.7	+
" directly after surgery	10.8	0.3	0.413	10.8	0.4	0.561	10.8	0.7	0.55
PEEP preoperatively	11.1	0.4	-	11.2	0.4	-	11	0.7	-
" 3 hours after surgery	10.8	0.4	0.347	10.7	0.4	0.246	10.9	0.7	0.973
PEEP preoperatively	11.1	0.4	-	11.1	0.4	-	11	0.7	+
" 6 hours after surgery	10.2	0.4	0.064	9.9	0.4	**0.025**	11	0.8	0.977
**Compliance** preoperatively	18.8	1.7	+	21.4	2.3	+	13.6	2	+
" directly after surgery	23.1	1.5	**<0,001**	25.2	2	**0.032**	19.1	2.3	**<0,001**
Compliance preoperatively	18.6	1.8	+	21.6	2.4	+	12.8	2	+
" 3 hours after surgery	21.6	1.6	**0.029**	23.9	1.9	0.238	17.3	2.3	**0.006**
Compliance preoperatively	18.6	14.3	+	21.7	2.5	+	12.8	2	+
" 6 hours after surgery	21	11.7	0.118	23.1	2	0.495	16.8	2.3	**0.011**
**Tidal volume** preoperatively	354.9	14.7	+	405.6	15.2	+	247.1	24.7	+
" directly after surgery	415.9	17.9	**<0,001**	454	9.7	**<0,001**	335.1	27	**0.001**
Tidal voume preoperatively	348.5	15.3	-	400.7	14.9	-	236	26.6	-
" 3 hours after surgery	315.2	18.3	0.085	362.3	18.8	0.081	213.6	35.2	0.567
Tidal voume preoperatively	346.4	0.3	-	401	15.8	-	236	26.6	-
" 6 hours after surgery	304.3	0.3	**0.033**	351.5	19.4	**0.032**	209	35.3	0.477
**Respiratory minute volume** preoperatively	7.2	0.5	+	8.8	0.4	+	4	0.5	+
" directly after surgery	8.3	0.4	**<0,001**	9.6	0.3	**0.012**	5.6	0.5	**<0,001**
Respiratory minute volume preoperatively	8	0.5	+	8.8	0.5	+	4.7	0.9	+
" 3 hours after surgery	9.2	0.4	**0.011**	9.5	0.5	0.098	7.9	1.3	0.053
Respiratory minute volume preoperatively	7.8	0.5	+	8.6	0.5	+	4.7	0.9	+
" 6 hours after surgery	9	0.4	**0.007**	9.3	0.4	0.082	8.1	1.2	**0.038**
**Mean arterial pressure** preoperatively (mmHg)	71.1	1.7	-	74.4	2	-	63.8	2.7	+
" directly postoperatively	69.9	1.3	0.528	71.4	1.5	0.2	66.6	2.4	0.361
**Heart rate** preoperatively (per minute)	94	2.2	-	99.7	2.4	+	81.4	4.3	+
" directly postoperatively	92.4	2.2	0.388	96.5	2.4	0.116	83.4	4.2	0.601
**SpO2** preoperatively (%)	90.7	1	+	90.1	1.4	+	91.8	1.4	+
" directly postoperatively	94.3	0.8	**0.005**	95	0.9	**0.003**	92.8	1.5	0.618
**FiO2** (%) preoperatively	65	2.3	+	61.6	2.8	+	71.1	3.6	+
" directly postoperatively	66.7	2.3	0.462	63.4	2.8	0.554	72.3	3.7	0.643
**Inspiratory pressure** preoperatively (mbar)	25	0.8	-	26.5	0.9	-	22.3	1.4	+
" directly postoperatively	24.6	0.7	0.387	25.8	0.8	0.286	22.4	1.3	0.902

Supplementary Table S2: Paired t-test for mean comparison of paO2/fiO2 ratio and lactate levels at different time points. We compared the preoperatively measured paO2/fiO2 ratio and lactate levels to postoperative values directly at different time points. This analysis was performed for all patients, non-ECLS patients, and ECLS patients.

All patients

		Mean	N=	p value
	preoperative	174		
			194	**p< 0.001**
	1h postoperatively	212		
	preoperative	178		
**paO2/fiO2**			191	**p=0.001**
	3h postoperatively	204		
	preoperative	177		
			183	**p<0.001**
	6h postoperatively	211		
	preoperative	7.2		
			197	**p=0.001**
	postoperatively	7.8		
	preoperative	7.0		
**lactate**			186	p=0.224
	6h postoperatively	7.3		
	preoperative	6.3		
			158	**p=0.040**
	24h postoperatively	5.6		

Non-ECLS patients

		Mean	N=	p value
	preoperative	171		
			134	**<0.001**
	1h postoperatively	205		
	preoperative	173		
**paO2/fiO2**			131	**0.006**
	3h postoperatively	195		
	preoperative	172		
			126	**<0.001**
	6h postoperatively	203		
	preoperative	6.7		
			133	**<0.001**
	postoperatively	7.4		
	preoperative	6.6		
**lactate**			126	0.186
	6h postoperatively	6.9		
	preoperative	6.0		
			112	0.200
	24h postoperatively	5.4		

ECLS patients

		Mean	N=	p value
	preoperative	182		
			60	**0.021**
	1h postoperatively	226		
	preoperative	189		
**paO2/fiO2**			60	0.066
	3h postoperatively	222		
	preoperative	188		
			57	**0.044**
	6h postoperatively	230		
	preoperative	8,5		
			64	0.724
	postoperatively	8,6		
	preoperative	7,9		
**lactate**			60	0.726
	6h postoperatively	8,0		
	preoperative	7,1		
			46	0.081
	24h postoperatively	5,9		

Supplementary Table S3: T-test to compare mean paO2/fiO2 ratio and lactate levels for survivors and non-survivors at different time points for all patients, non-ECLS patients, and ECLS patients

All patients

				**Survivors (n=64)**		**Non-Survivors (n=136)**		
		Median	Min/Max	Mean	Std.-deviation	Mean	Std.-deviation	p-value
paO2/fiO2-ratio	preoperative	155	36/570	183	101	174	125	**0.043**
	1h postoperatively	184	34/610	225	113	208	125	0.218
	3h postoperatively	176	45/543	214	106	200	119	0.289
	6h postoperatively	189	31/612	228	111	203	123	0.595
Lactate level / mmol/l	preoperative	6.2	0.4/28	4.4	3.9	8.6	5.5	**0.008**
	postoperatively	6.8	0.5/26	4.3	4.1	9.5	5.7	**0.002**
	6h postoperatively	5.7	0.5/27	3.9	3.8	9.0	5.8	**<0.001**
	24h postoperatively	3.1	0.5/26	2.1	1.3	7.8	5.9	**<0.001**

NON-ECLS patients

				**Survivors (n=52)**		**Non-Survivors (n=83)**		
		Median	Min/Max	Mean	Std.-deviation	Mean	Std.-deviation	p-value
paO2/fiO2-ratio	preoperative	155	36/498	191	91	157	98	0.576

	1h postoperatively	185	25/472	226	95	193	94	**0.009**
	3h postoperatively	176	45/480	220	96	179	80	0.193
	6h postoperatively	192	31/468	239	94	178	76	0.057
Lactate level / mmol/l	preoperative	5.4	0.5/28	4.2	3.9	8.2	5.7	**0.009**
	postoperatively	6.4	0.5/26	4.3	4.2	9.3	5.9	**0.014**
	6h postoperatively	4.9	0.5/27	3.9	4.0	9.1	6.0	**0.001**
	24h postoperatively	2.9	0.5/26	2.0	1.3	8.2	6.2	**<0.001**

ECLS patients

				**Survivors (n=12)**		**Non-Survivors (n=53)**		
		Median	Min/Max	Mean	Std.-deviation	Mean	Std.-deviation	p-value
paO2/fiO2-ratio	preoperative	132	40/570	149	134	202	156	0.342
	1h postoperatively	181	34/610	219	175	234	163	0.985
	3h postoperatively	172	46/543	188	142	234	158	0.332
	6h postoperatively	179	50/612	178	161	244	167	0.310
Lactate level / mmol/l	preoperative	8.3	0.4/24	5.5	4.1	9.3	5.1	0.441
	postoperatively	7.8	0.6/21	4.2	3.6	9.6	5.4	0.060
	6h postoperatively	7.5	1.2/21	4.0	2.8	9.1	5.4	**0.005**
	24h postoperatively	4.2	0.8/20	2.4	1.5	7.1	5.6	**<0.001**
